# Supplementary material for: Wafer-Scale Graphene Field-Effect Transistor Biosensor Arrays with Monolithic CMOS Readout
Source: ACS Appl Electron Mater. 2023 Aug 24;5(9):4925–32. doi: 10.1021/acsaelm.3c00706 (PMC10536967; doi:10.1021/acsaelm.3c00706)
Supplement: Supplementary file 1 — el3c00706_si_001.pdf [file el3c00706_si_001.pdf]

SUPPORTING INFORMATION

Wafer-Scale Graphene Field-Effect Transistor Biosensor Arrays with Monolithic CMOS Readout

Miika Soikkeli[1], Anton Murros[1], Arto Rantala[1], Oihana Txoperena[2], Olli-Pekka Kilpi[1], Markku Kainlauri[1], Kuura Sovanto[1], Arantxa Maestre[2], Alba Centeno[2], Kari Tukkiniemi[1], David Gomes Martins[1], Amaia Zurutuza[2], Sanna Arpiainen[1] and Mika Prunnila[1]*

[1]VTT Technical Research Centre of Finland Ltd, P.O. Box 1000, FI-02044 VTT, Espoo, Finland

[2]Graphenea Semiconductor SLU, Paseo Mikeletegi 83, 20009-San Sebastian, Spain

KEYWORDS: graphene, CMOS, monolithic, integration, wafer-scale, field-effect transistor, biosensor, statistics

Surface characterization

The surface of the graphene sensors was characterized by using an AFM to estimate the success of the channel opening processing step by measuring the roughness on the channel and the etch depth through the passivation stack. First the average roughness (R_a) of the CMOS wafers was measured to be used as a baseline value for the characterization (Figure S1a) Then the roughness on top of the graphene channel was measured from the middle of a chip (Figure S1b) and from the edge of a chip (Figure S1c) to estimate the amount of residue on top of the channel. The measured R_a values are 1.87 nm, 1.73 nm and 1.81 nm from the CMOS wafer before processing, middle of the chip final chip on graphene, edge of the final chip on graphene, respectively. This indicates that the channel opening has been uniform and there are no clear residues remaining from the processing steps. The etch depth of the passivation was also measured to estimate that the etching had reached graphene and gone through the Si_3N_4 and Al_2O_3 layers (Figure S1d-e). The etch depth corresponds to the total thickness of the Si_3N_4 (120 nm) and Al_2O_3 (100 nm) layers and this was ensured by overetching the Al_2O_3 layer with selective chemistry to graphene and underlying substrate.

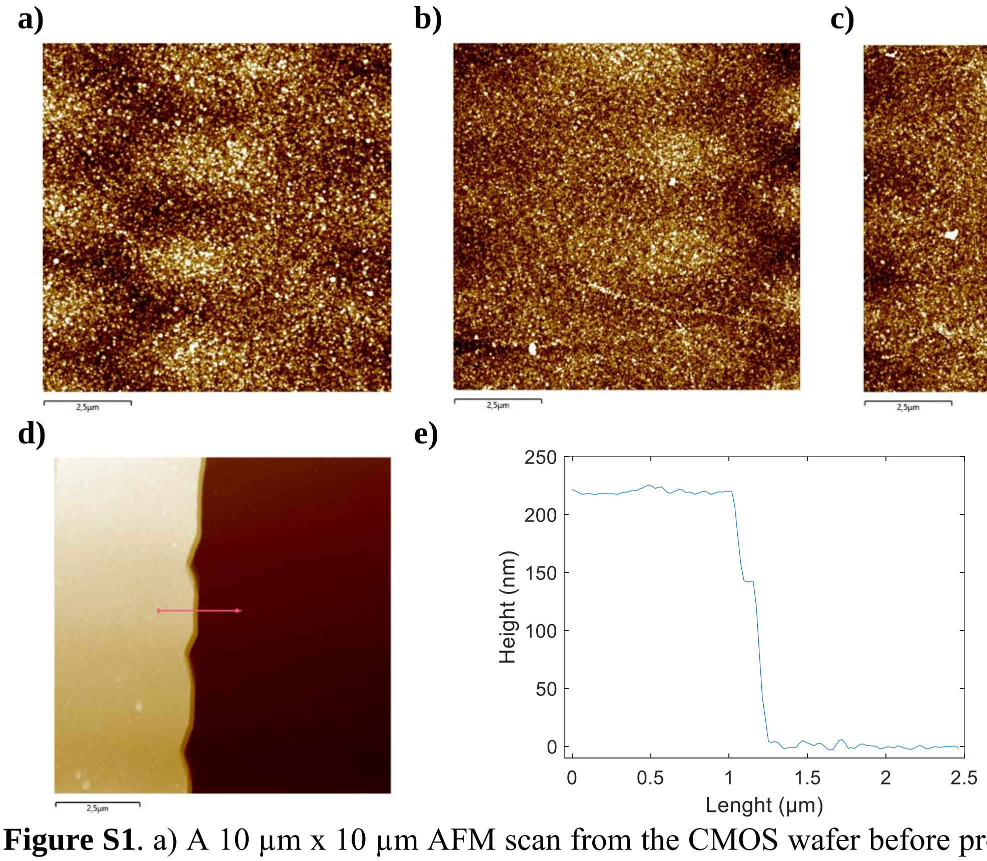

Figure S1. a) A 10 µm x 10 µm AFM scan from the CMOS wafer before processing with 10 nm data range. The measured R_a is 1.87 nm. b) a) A 10 µm x 10 µm AFM scan from the middle of the chip on graphene with 10 nm data range. The measured R_a is 1.73 nm. c) A 10 µm x 10 µm AFM scan from the edge of the chip with 10 nm data range. The measured R_a is 1.81 nm. d) a) 10 µm x 10 µm AFM scan from the edge of the passivation etching with 400 nm data range. The red arrow indicates the cross-section location in e) The etch depth of the passivation is about 220 nm which corresponds to total thickness of the Si_3N_4 and Al_2O_3 passivation layers.

Sensor array stability measurements

The stability of the sensor array was studied by measuring the response in DIW once before and after the actual measurement series including DIW 1, 1 mM NaCl, 10 mM NaCl and 100 mM samples. The Dirac peak voltage that has been normalized to the average value in DIW 1 changes from -9 mV (SD = 24 mV) in DIW_0 to 0 mV (SD = 13 mV) in DIW_1. The NaCl concentration series causes an average Dirac peak shift of 45 mV (SD = 15 mV), 82 mV (SD = 15 mV) and 129 mV (SD = 18 mV) for 1 mM, 10 mM, and 100 mM NaCl concentrations, respectively. The final measurement in DIW_2 shows an average of -13 mV (SD = 15 mV) Dirac peak voltage shift. The before and after measurements in DIW show that the baseline of the measurements only has minor changes between different measurements. The slightly higher standard deviation in the first DIW measurement is believed to be due to small amount of residues from the isopropanol cleaning and the additional rinsing with DIW before the DIW 1 measurement has improved the uniformity of the sensor array response. The slightly shifted Dirac peak position in DIW 0 and DIW 2 is also considered to be due to the isopropanol and acetone residues in the first case and NaCl residues in the latter one. This issue is believed to originate from the open cavity used in the measurements and could be improved with better fluidics integration to enable more efficient change of liquids in the cavity. Despite these small differences we can see that the baseline in DIW is clearly different and quite stable when compared to the Dirac peak voltage shifts induced by the NaCl concentration series.

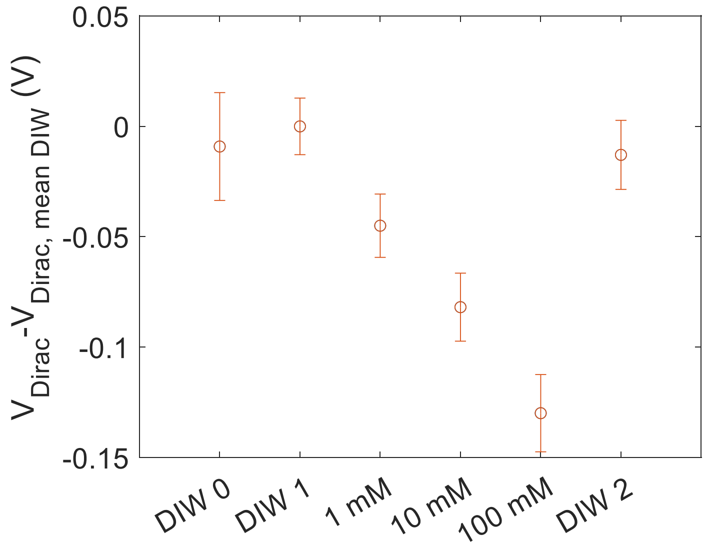

Figure S2. The average V_{Dirac}-$V_{Dirac, mean DIW\_1}$ values and standard deviations for the samples used to study the stability and the NaCl concentration series response.

Sodium chloride concentration series

The GFETs were characterized to obtain resistance and transconductance values of the devices as a function of the gate voltage for DIW and each NaCl concentration (Figure SI3). The average transconductance maximum and minimum values and standard deviations for the DIW, 1 mM, 10 mM and 100 mM NaCl concentrations are shown in Table S1. The resistance and the transconductance values indicate that the main response is the shift of the Dirac peak position. The transconductance minimum values on the hole carrier region remains nearly unchanged while the Dirac peak shift is clearly observed. There is a small change in the transconductance maximum values on the electron carrier region, but this asymmetry is likely caused by the higher voltage values needed to measure the electron carrier region.

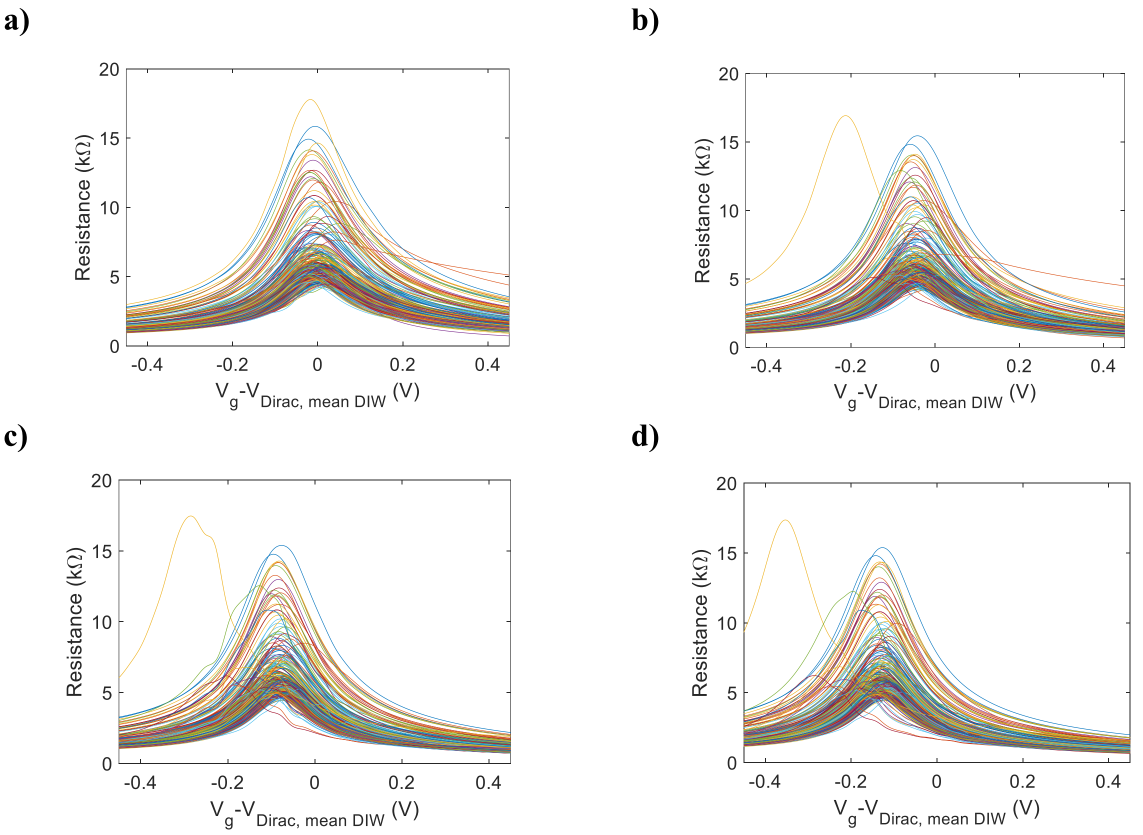

Figure S3. a) Resistance values as a function of the V_g-$V_{Dirac,mean\ DIW}$ for the 512 GFETs in DIW. b) Resistance values as a function of the V_g-$V_{Dirac,mean\ DIW}$ for the 512 GFETs in 1 mM NaCl. c) Resistance values as a function of the V_g-$V_{Dirac,mean\ DIW}$ for the 512 GFETs in 10 mM NaCl. d) Resistance values as a function of the V_g-$V_{Dirac,mean\ DIW}$ for the 512 GFETs in 100 mM NaCl.

Table S1. The average transconductance maximum and minimum values and standard deviations for the DIW, 1 mM, 10 mM and 100 mM NaCl concentrations.

	**gm_min,ave**	**gm_min SD**	**gm_max,ave**	**gm_max SD**
**DIW**	**-198 μS**	**33 μS**	**140 μS**	**33 μS**
**1 mM NaCl**	**-195 μS**	**32 μS**	**170 μS**	**36 μS**
**10 mM NaCl**	**-202 μS**	**34 μS**	**207 μS**	**35 μS**
**100 mM NaCl**	**-200 μS**	**34 μS**	**216 μS**	**35 μS**
